# Segmental patterning of microbiota and immune cells in the murine intestinal tract

**DOI:** 10.1080/19490976.2024.2398126

**Published:** 2024-09-10

**Authors:** Harithaa Anandakumar, Ariana Rauch, Moritz I. Wimmer, Alex Yarritu, Gudrun Koch, Victoria McParland, Hendrik Bartolomaeus, Nicola Wilck

**Affiliations:** a Experimental and Clinical Research Center, Cooperation of Charité-Universitätsmedizin Berlin and Max-Delbrück-Center for Molecular Medicine, Berlin, Germany; b Department of Nephrology and Internal Intensive Care Medicine, Charité - Universitätsmedizin Berlin, corporate member of Freie Universität Berlin and Humboldt-Universität zu Berlin, Berlin, Germany; c Max-Delbrück-Center for Molecular Medicine in the Helmholtz Association, Berlin, Germany; d DZHK (German Centre for Cardiovascular Research), Berlin, Germany

**Keywords:** Microbiome, immune cells, segmental patterning, germ-free vs colonized

## Abstract

The intestine exhibits distinct characteristics along its length, with a substantial immune cell reservoir and diverse microbiota crucial for maintaining health. This study investigates how anatomical location and regional microbiota influence intestinal immune cell abundance. Using conventionally colonized and germ-free mice, segment-specific immune cell composition and microbial communities were assessed. Metagenomic sequencing analyzed microbiome variations, while flow cytometry and immunofluorescence examined immune cell composition. Microbiome composition varied significantly along the intestine, with diversity and abundance increasing from upper to lower segments. Immune cells showed distinct segment-specific patterning influenced by microbial colonization and localization. T cell subsets displayed varied dependence on microbiome presence and anatomical location. This study highlights locoregional differences in intestinal immune cell and microbiome composition, identifying immune subsets susceptible to microbiota presence. The findings provide context for understanding immune cell alterations in disease models.

## Introduction

The intestine exhibits remarkable specialization along its length with distinct anatomical and functional characteristics. It serves as a crucial barrier for the host organism and provides a habitat for various microorganisms whose community structure changes along the intestine due to nutrient and chemical gradients, differing oxygen availability, transit rates, pH, and other factors. The intestine also harbors a substantial immune cell reservoir, the gut-associated lymphoid tissue (GALT), which likewise features a remarkable segment-specific organization and is exposed to various diet-derived antigens and commensal bacteria. As this exposure differs between segments, GALT composition, as well as the functional repertoire of the immune cells, changes.^1^ Prominent examples of the spatial organization of the immune-microbiome interaction include the effects of segmented filamentous bacteria (SFB) on the differentiation of T helper 17 cells (Th17) in the terminal ileum of mice^2^ and the influence of *Clostridium* strains in promoting regulatory T cells (Treg) in the colonic mucosa of mice.^3^

To elucidate the impact of bacterial communities on intestinal immune cells, germ-free (GF) mice provide a somewhat artificial, albeit valuable, experimental tool. Recently, GF mice have been used as non-microbiome controls to establish mechanistic links between commensals and diseases, among others, in allergic responses,^4^ immune response to chemotherapy,^5^ cardiovascular diseases,^6,7^ and motivation to exercise.^8^

Most studies that investigate the microbiome and associated immune phenomena tend to focus on specific cells or segments, neglecting the immune and microbiome compositions up- or downstream, potentially missing important information. Considering the length and compartmentalization of the intestine, phenomena observed in immune cells of a specific intestinal segment could differ in other segments. However, segment-specific immune cell abundances and phenotypes are insufficiently described even in healthy conditions. In a focused approach, studies have investigated immune populations of interest^9^ and their variation in GF and conventionally raised mice (CONV), with a specific-pathogen free (SPF) microbiota, at specific locations within the gut,^3,10–12^ while others have explored the variation of the gut microbiota along the gut.^13–15^ To the best of our knowledge, there is yet to be a coherent, single resource that maps the distribution of microbiota and immune cellular landscapes across the intestines of CONV and GF mice.

We used flow cytometry, immunofluorescence staining, and shotgun metagenomic sequencing to characterize the microbial and immune features along the intestine. We present this data as a resource to the community as an easy-to-explore and freely accessible app.

## Results

### Study overview

**Figure 1.**
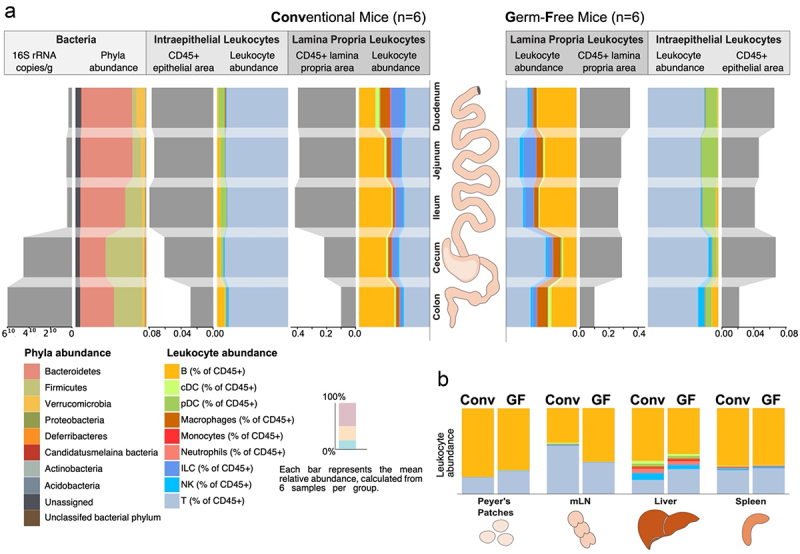
Overview of the segment-specific bacterial colonization and associated immune cells of the mouse intestine.

We explored the variability of the microbiota and immune cell composition across the intestine by analyzing conventional (CONV) specific-pathogen-free (SPF) C57BL/6J mice and germ-free (GF) C57BL/6J mice. The intestines were sectioned into five segments: three equally sized small intestinal segments, hereafter referred to as duodenum, jejunum, and ileum, as well as two large intestinal segments, the cecum, and the colon (Figure 1a and Figure S1a,b, Additional File 1). Measurement of 16S rRNA copies in the luminal contents by qPCR confirmed the GF status, as we did not detect any 16S rRNA copies (thus not shown). Additionally, GF mice displayed characteristic macroscopic changes, such as an enlarged and elongated cecum (Figure S1b, Additional File 1).

Abundances of bacteria and immune cells were shown for the five indicated intestinal segments: Peyer’s patches, mesenteric lymph nodes, liver, and spleen. (a) Illustration of absolute (gray) and relative (colored) quantities of intestinal bacteria, lamina propria, and intraepithelial leukocytes along the five intestinal segments of conventional (CONV, left) or germ-free (GF, right) mice. Bacterial taxa are shown at the phylum level as assessed by metagenomic sequencing. Absolute quantification of IEL and LPL by CD45+ immunofluorescence. Relative quantification of broad leukocyte subpopulations by flow cytometry. (b) The immune cell composition of Peyer’s Patches and mLNs as gut-associated organs, the spleen as a systemic lymphatic organ, and the liver as a central portal venous organ. Flow cytometry. CONV – Conventional mice, GF – germ-free, mLNs – mesenteric lymph nodes, PP – Peyer’s Patches. The bar plots represent the average relative abundance of each category across six independent samples per group (*n* = 6 mice per group).

To assess the microbiome’s taxonomic and functional variation, we conducted shotgun metagenomic sequencing on the luminal contents of CONV mice (Figure 1a). Apart from confirming previous findings^16–18^ where we noticed an increase in alpha-diversity from the oral to the aboral segments, we also noted that the proportion of relative abundance that could not be confidently assigned to any phyla decreased down the intestine (from roughly 8.2% to roughly 6.2%). This decrease occurred even though there was a significantly lower absolute abundance and diversity of bacteria in the small intestine compared to the large intestine.

Immune cell distribution along the intestines of GF and CONV mice was investigated by isolating intraepithelial leukocytes (IELs) and lamina propria leukocytes (LPLs) segment-specifically, followed by flow cytometry. The selection of LPL and IEL was based on their constant anatomical presence in each intestinal segment and their proximity to the gut microbiota. Other lymphatic structures like Peyer’s patches (PPs) can be mainly found in the aboral parts of the small intestine and were therefore pooled across the small intestine and analyzed for comparison. Broad immune cell subsets and their relative abundances per segment are shown along with CD45+ areas of IEL and LPL, quantified by immunofluorescence, in Figure 1a. Additionally, we explored the immune cell composition of other GALT-associated lymphoid compartments (mesenteric lymph nodes (mLNs) and PPs) as well as other lymphoid organs (spleen and liver) in GF and CONV mice (Figure 1b).

Within the lamina propria of CONV mice, a significant shift in the overall composition of immune cell subsets was evident along the intestine. Specifically, in the oral segments, innate immune cell subsets accounted for approximately 50% of the relative abundance, whereas in the aboral segments, there was a noticeable rise in the proportion of adaptive immune cell subsets, with innate subsets decreasing to approximately 20%. This organization of innate and adaptive cells along the intestine could not be observed in the LPL of GF mice. The IEL in both CONV and GF mice consistently exhibited dominance by T cells across all segments, comprising approximately 75% to 90% of the IEL population. However, variations in immune composition were also noted between CONV and GF in IEL, including an increased rise in NK cell abundance toward the colon of GF mice (Figure 1a).

The mLN and PP as gut-associated lymphoid organs showed differing immune cell compositions between GF and CONV (Figure 1b). The gut-associated immune cell profiles, assessed via principal component analysis (PCA), clustered samples based on the colonization status but also further strongly stratified them by their tissue origin, distinguishing between secondary lymphoid organs (mLNs and PPs) and LPL, emphasizing the dual influence of these factors on immune composition (Figure S1c, Additional File 1). Our data thus suggest that mLN and PP are substantially different from LPL and are an inappropriate proxy for LPL (Figure S1d, Additional File 1).

Large shifts in the immune composition caused by the colonization status were visible in the liver, with its portal venous blood supply, compared to only smaller shifts in the spleen as a systemic secondary lymphoid organ (Figure 1b). Overall, both the microbiome and immune cell composition vary broadly across segments (Figure 1a).

### Microbiota shape intestinal immune composition

**Figure 2.**
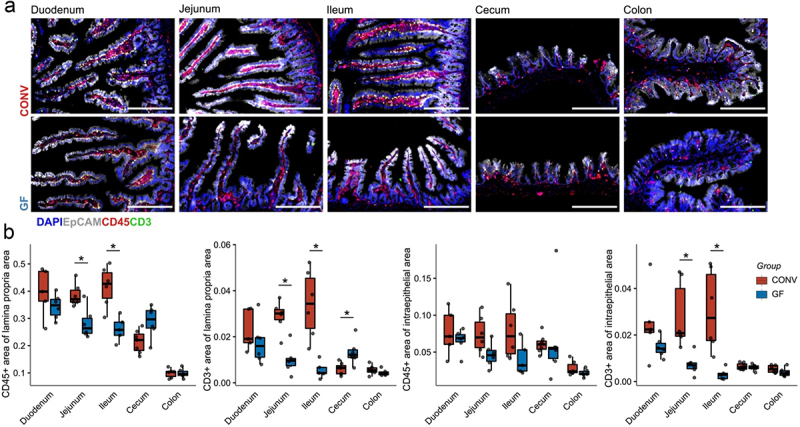
Absolute immune cell abundances and gut morphology in conventional and germ-free mice.

Developmental, functional, and structural differences in GF intestines have long been established.^19–21^ We aimed to spatially decipher the influence of the colonization status on the intestinal immune cell abundance and hypothesized T cells as one of the largest intestinal immune cell populations to be highly regulated by microbial colonization. To estimate the intestinal IEL and LPL quantities, we assessed the area occupied by leukocytes (CD45+) and T cells (CD3+) within the lamina propria or epithelium (EpCAM+) by immunofluorescence (Figure 2a and Figure S2a, Additional File 1). Unlike the enzymatic isolation of leukocytes from intestinal tissue with subsequent flow cytometry, which is prone to cell losses during processing, *in situ* immunohistological quantification provides an undistorted estimate of immune cell counts per segment. We confirmed previous findings^20^ of reduced epithelial and lamina propria areas in GF mice (Figure S2b, Additional File 1).^22^ We noted reduced leukocyte and T cell abundances in the lamina propria and intraepithelial areas of GF mice in the jejunum and ileum, albeit insignificant for the CD45+ IEL area (Figure 2b).

(a) Immunofluorescence analysis of GF and CONV intestinal sections. Sections were stained for CD45 (all leukocytes, red), CD3 (T cells, green), EpCAM (epithelium, gray), and DAPI (cell nuclei, blue). Representative images are shown. Scale bar equals 200 µm (b) Leukocytes and T cell abundance were quantified as the percentage of area covered within the lamina propria (defined as EpCAM negative) or epithelium area (EpCAM positive area). CONV are shown in red and GF in blue. CONV – Conventional mice, GF – germ-free. The p-values were calculated using Mann−Whitney U test and FDR corrected q-values shown (*q < 0.05) (*n* = 6 mice per group).

We conducted a comprehensive flow cytometry analysis for each GI segment to elucidate the immune landscapes across the intestine further. We quantified approximately 80 immune cell subsets (on a higher hierarchical level), the details of which are presented in the supplementary table (Table S1, Additional File 2).

**Figure 3.**
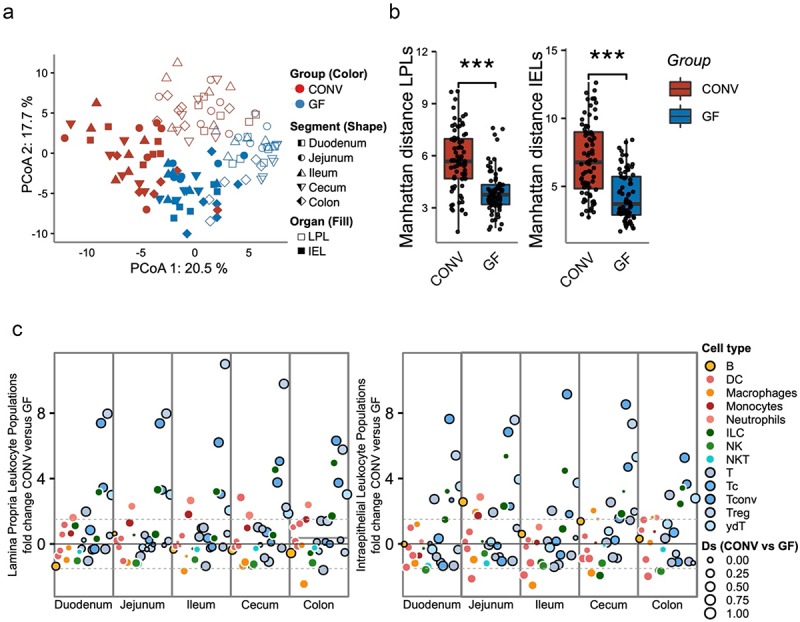
Intestinal immune cell clusters and composition of conventional and germ-free mice.

Considering the likelihood of comparable dietary antigen exposure in the oral segments between GF and CONV mice, we postulated that the colonization status might exert a more pronounced influence on immune cell populations in the distal segments compared to the proximal segments, given the higher microbial load in the distal segments. We observed that intestinal immune cells exhibited clustering based on their tissue of origin, whether IEL- or LPL-derived, as well as colonization status (Figure 3a). Contrary to our hypothesis, we did not observe distinct clustering patterns specific to individual GI segments, neither in the overall PCA (Figure 3a) nor in the PCA analyses restricted to IEL or LPL (Figure S3a-b, Additional File 1). Nevertheless, across all three PCA analyses (Figure 3a, Figure S3a-b, Additional File 1), the first principal component (PC axis 1) consistently captured the influence of colonization status, further underscoring its impact on immune composition. Notably, samples from CONV mice displayed a greater dispersion across the PCA space compared to GF mice. To quantify this dissimilarity, we calculated the distances between samples within and across all segments and noted that the distances within the CONV group were significantly higher for both LPL and IEL populations (Figure 3b). This observation may suggest that stochastic differences in individual microbial compositions contribute to variations in the immune phenotype.

(a) PCA of lamina propria (LPL) and intraepithelial (IEL) immune cell populations of GF and CONV mice from five intestinal segments. PCA was performed on z-score normalized relative immune abundances. (b) Inter-sample LPL and IEL distances across all CONV and GF mice intestinal segments. (c) Differential analysis of LPLs (left panel) and IEL (right panel) immune cell populations based on their different relative abundances in CONV and GF mice across all segments. Positive or negative fold change represents higher concentrations in CONV or GF, respectively. Black circles indicate that a certain cell population belongs to the adaptive immune system. The size of the circles represents the effect size of the difference between CONV and GF. The p-values were calculated using Mann−Whitney U test (***p < 0.001) (*n* = 6 mice per group).

Considering the well-established influence of gut microbiota on shaping the adaptive immune response, we posited that there would be a more pronounced regulation of adaptive immune cell populations, as opposed to innate immune subsets, when comparing GF vs CONV. Through univariate testing, we identified a collective reduction in the abundance of several adaptive immune populations, comprising 72 out of 138 tested populations (significant under FDR, p-value = 0.1) across all segments (in both IELs and LPLs, GF vs. CONV). Strong adaptive regulation was also noted at a more granular level of immune cell classification (Figure S3c, S3d, Additional File 1). Conversely, in the case of innate immune cells, we observed a change in 49 out of 130 tested populations under the same significance threshold, pointing to a comparatively less pronounced change. Furthermore, this effect on adaptive immune cells appeared to be independent of both the intestinal segment and the cellular origin (LPL vs. IEL) (Figure 3c), reaffirming the significant influence of colonization status on the clustering of immune subsets. Lastly, we could recapture higher levels of inter-sample distances in the CONV group across all segments when looked at in an origin-restricted manner (LPL or IEL) (Figure S3e−f, Additional File 1).

Taken together, our findings reveal the interplay between colonization status and immune cell variations specific to different segments. Notably, within CONV mice, the microbiome’s mere presence emerges as a potent contributor to increased inter-individual variance. This underscores the substantial impact of colonization status on immune composition throughout the intestine. In essence, segment-specific variations are modulated by microbial presence, highlighting the complexity of immune cell patterning in the intestine.

### Spatial organization of gut microbiota

**Figure 4.**
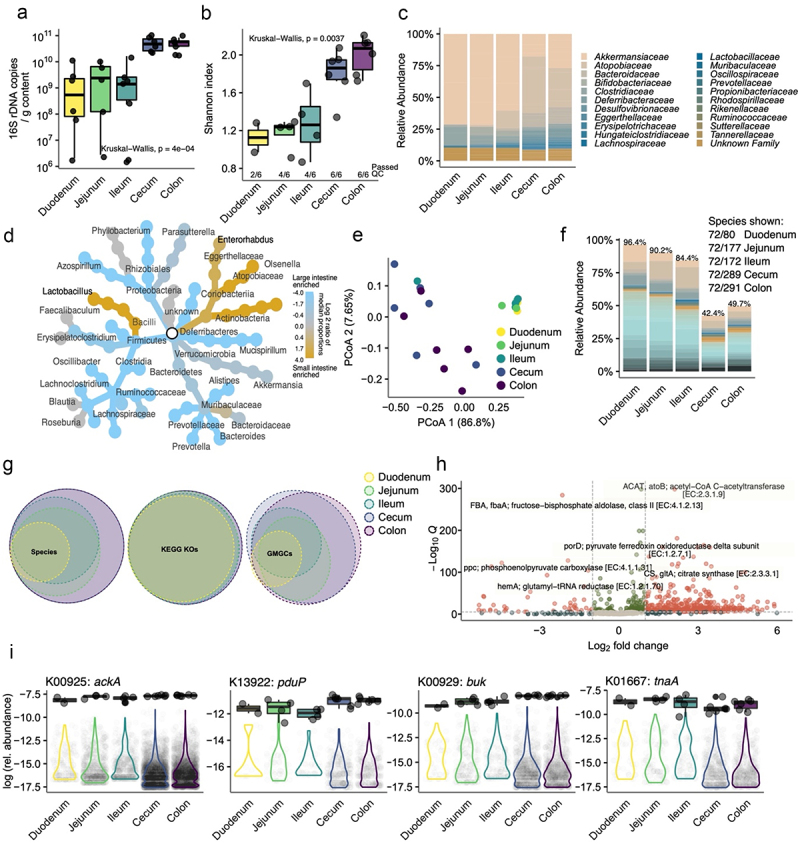
Microbiome variation along the small and large intestines of mice.

Intestinal characteristics extend beyond immunological and anatomical features, encompassing changes in the composition and function of the gut microbiome. To explore the microbiota representative of the different intestinal segments of CONV mice, we performed shotgun metagenomic sequencing of the respective luminal contents. We confirmed a significant increase in bacterial loads from the oral to the aboral end, ranging from ~10^8^ 16S copies per g of content in the duodenum to ~10^11^ 16S copies per g of content in the colon (Figure 4a). This increase was accompanied by an increase in the complexity and diversity of the bacterial composition (Figure 4b,c). To easily visualize taxonomic differences, differential heat trees were constructed showing differences between large intestinal (colon and cecum) and small intestinal (duodenum, jejunum, and ileum) samples at the genus (Figure 4d) and species (Figure S4a) level. To further assess the diversity of the microbial profiles of the intestinal segments, we performed a beta diversity analysis on Bray‒Curtis distances at the species level (Figure 4e). Not surprisingly, the small intestinal segments clustered tightly, with the cecal and colonic samples clearly distinctly clustered. Interestingly, in the cleaned and filtered relative abundance dataset at the species level, we observed that most of the species (72/80) found in the duodenum persisted across the entirety of the intestine when seen in a binary presence-absence manner (Figure 4g, left-most venn diagram). However, the number of uniquely detected species, also a measure of richness, drastically increased along the intestine from 80 unique species in the duodenum to 291 in the colon. Despite accounting for only 20% of the detected species in the large intestinal segments, these 72 segment-overlapping species made up ~50% of the relative abundance in the large intestinal segments (Figure 4f). This indicates that a small number of bacterial species persist at a relatively high abundance from the oral to the aboral intestinal segments of mice.

The intestinal contents along the intestines of CONV mice were collected. (a) 16S rRNA copies per g of luminal content quantified by qPCR (*n* = 6 per segment). (b) Alpha diversity was measured by Shannon index from species-level taxonomic annotation of shotgun metagenomic sequencing. (c) Bar plots showing bacterial family-level taxa along the different GI segments. (d) Differential heat tree depicting the taxonomies of bacterial genera that significantly differed in their relative abundance between small (*n* = 10) and large intestinal (*n* = 12) samples. Blue colored nodes show enrichment of those taxa in the large intestine. (e) Beta diversity is shown as PCoA using the Bray−Curtis dissimilarity matrix indicating distinct small and large intestinal clustering. (f) The relative abundances of the 72 species detected in all segments. Bar plots indicate relative abundances. Absolute numbers of detected species are shown in the right. Percentages on top of bars show the proportion of the added abundance of 72 common species in relation to total species abundance. (g) Euler diagrams indicating the number and overlap of detected species across the different segments. Euler diagrams for the (binary) presence of functional genes are annotated using Kegg orthologues (KOs) or global microbial gene catalogs (GMGCs). (h) Volcano plot indicating enrichment of KOs in the small intestine (to the left, *n* = 8) or large intestine (to the right, *n* = 12). The Y-axis indicates that the q-value, and the X-axis, log-transformed fold changes. (i) Abundance of representative KOs are shown as boxplots across segments (data points as black circled gray dots), and abundance of corresponding GMGCs are shown as violins (pale gray dots in background). From left to right: acetate kinase (ackA), propionaldehyde dehydrogenase (pduP), butyrate kinase (buk), and tryptophanase (tpaA). Data in B, C, E−G, I: duodenum *n* = 2, jejunum *n* = 4, ileum *n* = 4, cecum *n* = 6, and colon *n* = 6. For statistical comparison, duodenum samples (only *n* = 2 from six samples passed QC) were excluded.

Despite the stark variation in the absolute number of unique bacterial species detected across the intestinal segments, at the functional level assessed by KEGG orthologs (KOs), we observed an almost complete overlap between the segments when analyzed simply for the presence or absence of each KO (Figure 4g, center venn diagram). However, when analyzed at the level of prokaryotic genes that make up these KOs, annotated as genes from the Global Microbial Gene Catalog (GMGCs), we noticed the reappearance of clear segment-specific functional patterns resembling the species-level variation (Figure 4g, right-most venn diagram), where only ~31% of GMGCs were detected commonly across all segments, the overlap for KOs was ~82%. In line with the literature,^23,24^ we detected large-scale shifts in the abundance of functional pathways when comparing small versus large intestine (Figure 4h, Figure S4b, Additional File 1). This data suggests that as we go down the intestine, there is an increase in the diversity and richness of the bacterial species and the GMGCs mapped to each sample, while the presence of KOs is mostly constant.

Interestingly, we observed a higher number of unique GMGCs attributed to a given KO in the large intestinal segments compared to the small intestine, likely due to increased species diversity. However, this pattern did not hold when examining the average relative abundance of these KOs. Instead, it appeared that the relative abundance of a specific KO remained relatively stable across the entire intestine, while the number of unique GMGCs or genes associated with them increased (Figure S4c, Additional File 1). For example, KOs related to acetate metabolism, such as KO:K00925 (acetate kinase, ackA), exhibited this pattern (Figure 4i).

However, when examining other KOs specific to short-chain fatty acid (SCFA) metabolism, a different pattern emerged. The KO:K13922 (propionaldehyde dehydrogenase, pduP) and KO:K009292 (butyrate kinase, buk), responsible for propionate and butyrate formation, respectively, showed an increase in both relative abundance and the number of unique GMGCs down the GI tract, consistent with the known high abundance of SCFA in the large intestine. Similarly, functions attributed to the upper GI tract, for example, bacterial indole formation from tryptophan (KO:K01667, tryptophanase, tnaA), exhibited the highest relative abundance in the oral segments despite having more unique GMGCs in aboral segments (Figure 4i).

Collectively, our findings extend the existing knowledge that murine microbiome diversity at the species or gene level does not necessarily correlate with functional-level diversity throughout the intestine. While average patterns can be ascertained, our results highlight region-specific and function-specific variations in microbial functionality.

### Immune population-specific patterns of microbiome-immune interactions

**Figure 5.**
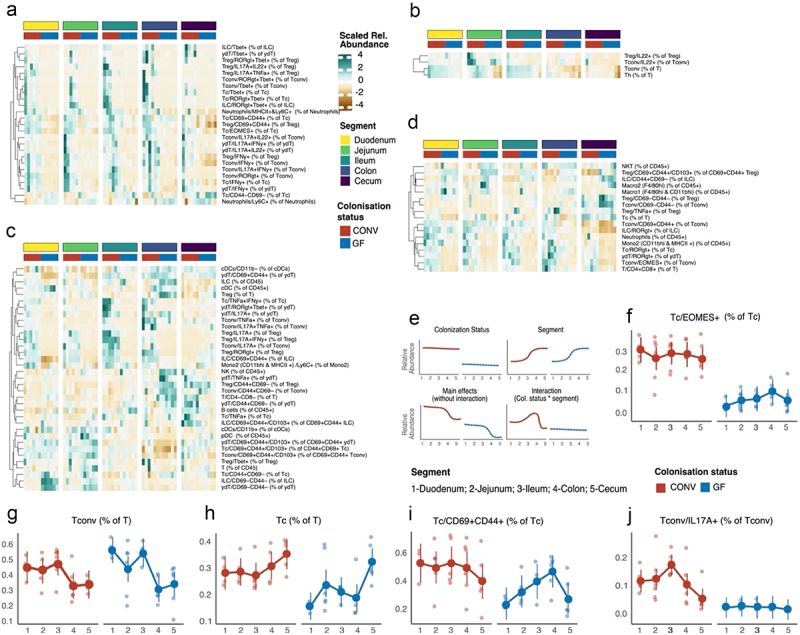
Influence of microbial colonization and segment on the abundance of intestinal immune cells.

Following the description of immune and microbiota variability along the intestine in GF and CONV mice, we sought to determine which immune cell distributions exhibited a pattern influenced by the presence of bacteria rather than by the intestinal segment-specific anatomical factors. We used linear models as a tool to discriminate immune cell patterns into groups whose relative abundance is best modeled by either the colonization status or the segment of origin, their interaction, or both without interaction. Hence, we categorized LPL immune populations according to the factors that best modeled their abundance: the colonization status (25/82, Figure 5a), a combination of both segment and colonization status (17/82, Figure 5b), or their interaction (36/82, Figure 5c) or only the segment (4/82, Figure 5d). We conducted an enrichment analysis using a one-sided Fisher’s exact test to determine whether immune populations were overrepresented in these categories. At a broad level, we found that dendritic cells, CD3+CD4+ Th cells, and CD3+CD8+ Tc cells were enriched in groups dependent on interactions, intestinal segments, and colonization status, respectively (p-values: 0.024, 0.044, and 0.074). When we examined specific/granular immune cell phenotypes, we observed the enrichment of cell types, such as those expressing markers for RORgt+Tbet+, Tbet+, IFNy+, and IL17A+IL22+ in the colonization status group. In contrast, IL22+ cell types were enriched in the segment group, while cDC, IL17A+, CD69+CD44+CD103+ (tissue-resident T-cell subtypes), and CD44+CD69- (memory T cells) cells were enriched in the interaction group. The distribution patterns of the immune cells in the different segments in relation to these factors can be visualized, as schematically illustrated in Figure 5e. For example, cytotoxic T cells that express eomesodermin (EOMES) were dependent on colonization status in the absence of a strong regional organization (Figure 5f). Similarly, the overall percentage of conventional T cells among all T cells was only dependent on the segment in both CONV and GF mice (Figure 5g). In contrast to these populations, which are highly dependent on the segment-specific effect or only on the colonization status, other populations were found to be dependent on both factors in a non-interacting manner, for example, the regional abundance of cytotoxic T cells among all T cells (Figure 5h). Lastly, we want to highlight here tissue-resident cytotoxic T cells, as an example of an immune cell population whose abundance is best modeled by the interaction of segment and colonization effect, where we observed a spatial organization in GF mice, with a peak in abundance in the cecum, which was lost under microbial colonization (Figure 5i). This indicates that the mere presence of microbial antigens across the intestine can sometimes override the immune spatial organization, as in the case of GF mice. To investigate potential associations between specific gut microbial species and immune cell subsets (LPLs), we performed correlative analyses using both absolute and relative abundance data of microbiome species level and immune cell data (Figure S4d, Additional File 1). Despite the inherent limitations of correlative approaches,^25^ we observed consistent links between certain immune cell subsets and microbial species. RORγt+ conventional T cells exhibited correlations with species from the Lactobacillus and Alistipes genera, while innate lymphoid cells (ILCs) were associated with the Oscillibacter species and members of the Lachnospiraceae family.

The dependence of the abundance of various immune cells on localization in the intestine, colonization, or both factors (interaction) was tested by linear modeling. K-means clustered heatmaps (a−d) show the abundance of the different immune subsets per segment, which were best modeled by colonization status (a), intestinal segment (d), or both factors without (b) or with (c) statistically significant interaction. (e) Schematic illustration of the identified immune cell patterns and their different categories (4). Per category, the abundance of the immune cell population in CONV (left, red) and GF (right, blue) is shown across intestinal segments. f−j shows representative immune cell populations for the four patterns mentioned above. (f) Distribution of EOMES expressing cytotoxic T cells as a representative population for colonization influence. (g) The relative abundance of conventional T cells within T cells as a representative for segment influence. (h) Cytotoxic T cells as a representative for both main factors becoming significant without interaction. (i) Resident memory status (CD44+CD69+) within cytotoxic T cells and (j) IL-17A expression within conventional T cells and as representative populations for the interaction of colonization and segment influence. Solid points and bars indicate the estimated marginal means from the model overlaid on real, alpha-d data points. Abbreviations: T: T cells, Tc: cytotoxic T cells, Th: T helper cells, Tconv: conventional (FoxP3-) Th, Treg: regulatory (Foxp3+) T cells, γδT: gamma delta T cell, ILC: innate lymphoid cells, Mono: monocytes, DC: dendritic cells, NK: natural killer cells (*n* = 6 per segment).

One other compelling illustration of the interplay between the microbiome, immune responses, and spatial organization is seen in the context of IL-17A production. Our study notably identified a microbiome-dependent skew toward IL-17A-producing T helper cells (Figure 5j), specifically enriched in the ileum (categorized under the interaction-dependent immune cell population category). Intriguingly, our shotgun metagenomic data revealed the presence of segmented filamentous bacteria (SFB) in our CONV mice (Figure S4e, Additional File 1), with their presence only detected and notably elevated in the ileum. SFB, known for its potent influence on the Th17,^2^ is a prime example of microbiota driving immune responses in a segment-specific manner. The spatial enrichment of SFB in the ileum aligns with the heightened IL-17A production in conventional T helper cells observed in the same region.

**Figure 6.**
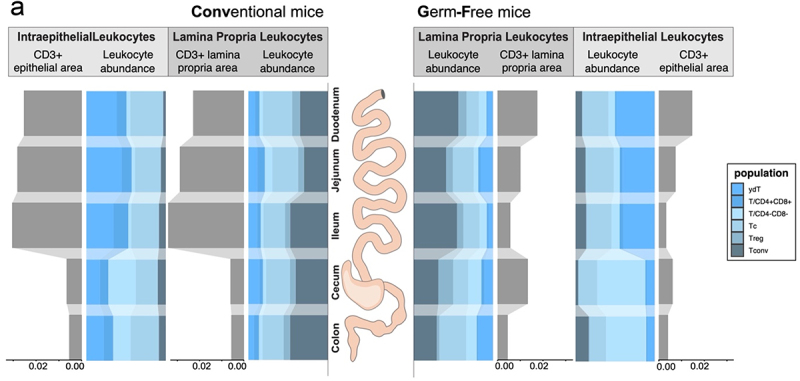
Summary of the segment-specific T cell distribution across the mouse intestine.

Taken together, the spatial organization of T cell subsets shows the strongest regulation by microbiota in our data. We summarized our findings on the major T cell subpopulations in the LPL and IEL of GF and CONV in Figure 6.

(a) Distribution of absolute (gray) and relative (colored) changes of lamina propria and intraepithelial T cells along the different segments of the intestine in conventional (CONV) and germ-free (GF) mice (*n* = 6 per segment).

In summary, our study explored the locoregional differences in immune cell and microbiome composition of the intestine and identified immune subsets susceptible to and influenced by the mere presence of microbiota. Lastly, we provide all our comprehensive immune and microbiome data as an open-access app for exploration: https://wilck-lab.shinyapps.io/galt_2024_shiny/. This resource could aid in contextualizing changes in immune cells across various disease models.

## Discussion

The remarkable diversity of the gut microbiome across different segments of the intestine underscores the interplay between microbial communities and their host environment. Our study revealed distinct patterns of microbial colonization along the intestine. This spatial distribution aligns with known physicochemical parameters that characterize each segment, such as pH, oxygen availability, and nutrient gradients.^23^ These findings corroborate the notion that microbial communities have adapted to thrive in the unique microenvironments offered by different gut segments.

Moreover, our analysis of immune cell composition provided compelling evidence for segment-specific immune patterns. The dominance of different immune cell subsets, both in the IEL and LPL compartments, varied along the intestine. The interaction between the microbiome and segment-specific immune responses appears to be reciprocal. The microbiota not only adapts to the local conditions of each segment but also influences the composition and function of immune cell populations, as shown here for various T cell subsets. This dynamic cross-talk suggests that local microbial communities might be contributing to driving segment-specific immune adaptations. Similarly, the immune system’s regulation of microbial communities likely impacts the selection and survival of certain microbial species. Research has demonstrated that alterations in microbial community diversity and composition along the gastrointestinal tract are associated with the pathogenesis and severity of various diseases, including inflammatory bowel disease (IBD).^26^ In IBD, reduced microbial diversity and increased abundance of Enterobacteriaceae in the ileal mucosa have been shown to correlate with inflammation and disease severity.^27^ Additionally, the expansion of oral-specific microbes, such as *Veillonella parvula*, in the intestines of IBD patients has been linked to disease progression.^28^ These findings highlight the importance of maintaining the niche-separation of the microbiota along the gastrointestinal tract and the potential consequences of its breakdown in diseased states.

### Microbiome-immune map as a future resource

Our investigation into microbiome-immune interactions revealed insights into the crosstalk between these two systems. This mutual influence shows that the gut microbiome is not merely a passive inhabitant; it actively contributes to immune cell diversity and function. Recent studies have begun to shed light on the dynamic crosstalk between the gut microbiome and the immune system over time in various clinical settings. A notable example is a longitudinal study that followed cancer patients receiving immune checkpoint blockade (ICB) therapy.^29^ Using daily measurements of neutrophil, lymphocyte, and monocyte counts along with longitudinal gut microbiome samples, they found consistent associations between specific gut bacteria and immune cell dynamics during immune reconstitution post-transplant, providing direct evidence in humans that the gut microbiota can modulate systemic immune cell dynamics, with potential implications for augmenting immune-targeted therapies.

Our study corroborates the notion of distinct niches across the gastrointestinal tract, characterized by heterogeneous microbial communities and immune cell distributions.^30^ While our results on taxonomic alterations along the gut are generally consistent with prior studies,^15,17,18,31–33^ we observed some discrepancies in alpha diversity metrics derived from 16S rRNA gene sequencing datasets in other studies.^14,34^ These differences may be attributed to the limitations of comparing 16S and shotgun metagenomic sequencing approaches, as well as reasons discussed by Li et al.^17^ The concept of a ‘core’ microbiome consistently present along the length of the intestine is not new.^35^ Although sampling technical artifacts could potentially contribute to this observation, our samples clustered based on origin (either the large or small intestine) and the inclusion of blank control samples that did not pick up bacterial signatures similar to any of the segments suggests a definitive biological signal. Additionally, coprophagia in mice is a known phenomenon,^36,37^ which could lead to fecal-oral-fecal transmission and further contribute to the detection of the same microbial species across all gut segments. The identified segment-specific patterning of immune cells influenced by both microbial colonization and anatomical localization echoes the intricate metabolomic interplay previously published.^30^ Integrating these datasets may contribute to a deeper understanding of the complex interactions between the microbiome, immune system, and metabolome along the gastrointestinal tract. However, a lack of high-quality, publicly accessible data from different studies prevented us from performing direct comparisons, underscoring the importance of open data sharing policies in advancing our understanding of these complex systems.

Our analysis of immune cell subsets demonstrated a differential dependence on microbiome presence and gut segment. Notably, the microbiome is highly influenced by T cell subsets, such as Th17 cells and cytotoxic resident memory T cells. In contrast, overall conventional T cells showed stronger regulation by their spatial distribution along the intestine. These findings imply that certain immune populations might be more susceptible to microbiome-driven modulation. Several reviews have covered specific interactions between different immune cell subsets and microbial species in depth.^38,39^ Furthermore, the presence of the microbiome itself appears to introduce an additional layer of variability in immune cell composition, leading to increased inter-individual variance. This implies that for immunologically focused research, authors should consider at least biobanking the microbiome. In line with this, it is becoming common for studies to use replication across animal facilities as an additional level of evidence or identify microbiome differences as potential sources for non-reproducible findings.^40,41^ Studies have demonstrated that GF mice possess a functional, albeit altered, immune system capable of responding to microbial colonization. Our current data show that while the immune composition in GF mice is indeed skewed compared to conventional mice, there exists a substantial and diverse immune cell repertoire in various organs, including the spleen, providing an adequate representation of overall immune status.

In general, naïve T cells differentiate upon antigen exposure into effector and memory subsets. Our dataset revealed that tissue-resident T cells (TRM), an interesting subset, are regulated by the interaction of microbiota with specific intestinal segments. Recent studies have linked the training of TRM during polymicrobial sepsis to the antitumoral effects of these cells.^42^ Our data indicate that TRM training also occurs in a microbiome-dependent manner under physiological conditions, which is in line with several studies implicating gut microbiota in tumor and metastasis growth.^43^ Future studies are needed to investigate which microbiota and metabolic pathways most effectively train TRMs. In addition to conventional T cells, Tregs exhibited altered memory and tissue residency phenotypes under microbial colonization. Treg also displayed a distinct cytokine and transcription factor profile, including Th17-like phenotypes. These effector-like Tregs are described in humans and are linked to a distinct pool of antigen-specific T cell receptors.^44^ Several studies suggest they are dysfunctional,^45,46^ while others propose they are highly suppressive due to homing via shared chemokine receptors.^47,48^ Again, our data highlights how distinct microbial colonization across the GI tract influences these cells and future research is needed to identify specific microbes or metabolites. The suppressive capacity of Treg is of importance in a disease-dependent manner; while cardiovascular disease and autoimmunity are characterized by dysfunctional Treg, a higher amount of suppressive Treg in the tumor microenvironment plays a prominent role in tumor development. Lastly, we highlighted EOMES expressing Tc as microbially induced. These cells exhibit strong antitumoral effects,^49^ further emphasizing the role of gut microbiota in cancer.

While it is beyond the scope of the current project to investigate the influence of age and sex on the immune-microbiome dynamics, we acknowledge that one of the main limitations of the study is that it was performed only in male mice. Emerging evidence^38,50,51^ emphasize the need to explore age- and sex-dependent variations in immune-microbiome relationships, offering an important direction for future research in the field of host–microbe interactions. Additionally, efforts like the Tabula Sapiens project^52^ and others^53^ have started to map the cellular landscape of the human gut at high resolution using single-cell approaches. However, integrating this information with microbiome profiling remains an important next step to unravel the complex interactions between the gut microbiome and host immunity.

To facilitate accessibility and further exploration of our findings, we have developed a user-friendly app. The app allows researchers to delve into specific immune categories and their interactions with microbiome profiles, fostering deeper investigations into the mechanisms underlying the observed effects. Additionally, the open-source data enable the broader scientific community to validate and expand upon our findings, potentially uncovering novel associations and mechanisms that contribute to the dynamic interplay between the microbiome and the immune system.

### IL-17A production and microbiome dependency: a key example of spatial regulation

One of the best-described associations between microbiota and immune cells is Th17 cells, with their peak abundance in the ileum. This association is again found in our data, suggesting that SFB contributes to the local immune landscape. This effect was shown to be dependent on direct interactions of the gut epithelial cells with these bacteria.^54,55^ The correlation between SFB abundance and IL-17A production not only underscores the microbiome’s role in shaping specific immune phenotypes but also exemplifies the intricate coordination between microbial colonization and immune regulation along the gastrointestinal tract. However, IL-17A production was observed across multiple immune cell types with varying abundances along the intestinal tract, indicating additional microbiome factors likely influence Th17 polarization.

While our study offers valuable insights into the complex interplay between the microbiome and immune system along the intestine, it is essential to acknowledge the inherent limitations of our T cell-centric phenotyping panels. By primarily focusing on T cells, we potentially missed insights into complex interactions involving innate immune cells, antigen-presenting cells, and regulatory populations. Moreover, the characterization of certain cell populations is constrained by the limited set of surface markers employed in our panels. While our approach provides a broad overview of immune cell composition, it may not definitively identify all cell subtypes with the precision afforded by more extensive marker panels. This limitation is particularly relevant for complex populations such as ILCs and NKT cells, which typically require additional markers for unambiguous identification. To mitigate this limitation, future investigations should expand phenotyping panels to encompass a broader array of immune cell subsets and leverage multi-omics approaches. Additionally, it is important to note that our study investigated the microbiome-immune interplay within one specific mouse-strain-microbiome context. Extrapolating findings to different microbiome compositions and/or facilities warrants careful consideration due to potential variations in species prevalence, functional potential, and immune responses, all of which may influence segment-specific dynamics.

## Materials and methods

### Mice

All data shown were generated following German/European law for animal protection. Only healthy, untreated, conventionally colonized, and germ-free mice were used. A license for the use of the animals for research purposes was granted (X9011/21). Eight-week old wild-type male conventionally, colonized SPF C57BL/6J mice (CONV, *n* = 6) were purchased from the Charles River (Europe Mouse SPF/VAF Barrier Room – Sulzfeld A004 Mice) and were kept for 2 weeks in-house. Ten-week-old wild-type male germ-free C57BL/6J mice (GF, *n* = 6) were received from an in-house axenic breeding (Charité, Berlin, Germany). An overdose of isoflurane was used to euthanize these 10-week-old male mice. Sample size was not a priori calculated and was chosen from experience for exploratory analysis. Subsequently, the abdomen and thorax were opened, the right atrium was cut open, and the mice were flushed with NaCl from the left ventricle until the liver was discolored. Luminal contents were collected before flushing, and mice were in a fed state upon sacrifice.

### Organ collection, immune cell isolation, and flow cytometry

Organ collection was performed from six animals per group. Spleens and mesenteric lymph nodes were dissected and kept in PBS/0.5% BSA/2 mM EDTA at 4°C. The right kidney was decapsulated and kept in PBS/0.5% BSA/2 mM EDTA at 4°C. One lobe of the liver was dissected and kept in PBS/0.5% BSA/2 mM EDTA at 4°C. The intestines were flushed with HBSS (without Ca2+ Mg2+)/10 mM HEPES to remove leftover feces, divided into 5 sections (3 sections of the small intestine, cecum, and colon), and stored in HBSS (without Ca2+ Mg2+)/10 mM HEPES at 4°C. Peyer’s patches were dissected from the small intestinal sections and kept in PBS/0.5% BSA/2 mM EDTA at 4°C. Single – cell suspensions of splenocytes were obtained by pressing the tissue through a 70 µm strainer, washing with PBS/2 mM EDTA, erythrocyte lysis (83 g/l NH4Cl/8.5 g/l NaHCO3/10 mM EDTA), and further filtering with a 40 µm strainer. Mesenteric lymph nodes and Peyer’s patches were punctured, flushed with PBS/0.5% BSA/2 mM EDTA, and filtered through a 70 µm strainer. The cell suspensions of the spleen, mesenteric lymph nodes, and Peyer’s patches were counted using the LUNA-FL Dual Fluorescence Cell Counter (logos Biosystems). Kidney and liver cell suspensions were obtained with mechanical and enzymatic dissociation (1.14 mM collagenase IV and 255 mM DNase I in HBSS with Ca2+ and Mg2+/10 mM HEPES/5% FBS) via a MACS dissociator (Miltenyi Biotec). The digestion was stopped with PBS/10% FBS, followed by erythrocyte lysis (83 g/l NH4Cl/8.5 g/l NaHCO3/10 mM EDTA). The cell suspension was then cleaned by filtering through a 70 µm strainer, 40%/80% Percoll density gradient centrifugation, and final filtering through a 40 µm strainer using PBS/0.5% BSA/2 mM EDTA. Mechanical and enzymatic dissociations were also obtained intestinal cell suspensions of each segment’s lamina propria and intraepithelial lymphocytes. The Mouse Lamina Propria Dissociation kit (Miltenyi Biotec) was used according to the manufacturer’s protocol. The cell debris was cleaned by filtering through a 100 µm strainer, 40%/80% Percoll density gradient centrifugation and subsequent filtering through a 40 µm strainer using PBS/0.5% BSA/2 mM EDTA.

The isolated cells were either directly stained for flow cytometric analysis or restimulated with 50 ng/ml phorbol-12-myristate-13-acetate (PMA, Sigma‒Aldrich), 500 ng/ml ionomycin (Sigma‒Aldrich), and 0.75 μl/ml GolgiStop (BD Bioscience) for 4 h at 37°C and 5% CO2 in RPMI 1640 medium (Sigma‒Aldrich) supplemented with 10% FBS and 1% penicillin – streptomycin. For flow cytometry analysis, dead cells were labeled with a Live/Dead Fixable Aqua Dead Cell Stain Kit at 405 nm excitation (Thermo Fisher), followed by surface antibody staining (Table S2, Additional File 2) in PBS/0.5% BSA/2 mM EDTA together with Fc blocking reagent (Miltenyi). Cells were first permeabilized for intracellular staining and then stained with intracellular antibodies using the FoxP3 Staining Buffer Kit (eBioscience). All steps were performed for 30 min at 4°C. The data were recorded on a BD LSRFortessa Cell Analyzer using BD FACS Diva software (BD Bioscience) and analyzed using FlowJo 10.8.1 (BD Bioscience).

### Immunofluorescence and histological staining

Tissue pieces from the five intestinal sections were dissected. The tissues were fixed for 5 h using 4% EM-grade PFA (Electron Microscopy Sciences) on PBS at 4°C, washed in PBS, cryoprotected using a sucrose gradient (15% 3–6 h, 30% 17 h), and frozen in Tissue-Tek O.C.T. compound (Sakura Finetek 12,351,753) using −40°C 2-methylbutan. Tissues were cut into 7 µm cryosections and stored at 80°C.

For immunofluorescence staining, sections were rehydrated in TBS and permeabilized with TBS/0.1% Tween, and nonspecific binding was blocked with TBS/0.1% Tween/10% normal donkey serum (NDS)/5% normal goat serum (NGS)/5% bovine serum albumin (BSA) for 20 min at RT. Staining was performed with antibodies in TBS/0.1% Tween/5% NDS in a humid chamber. Primary antibodies (anti-CD45, 30-F11 (RUO), BD Pharmingen 553,076, 1:100; anti-CD3, 145-2C11, StemCell 60,015, 1:100; anti-EpCAM, E144, Abcam, ab32392, 1:100) were incubated overnight at 4°C. Primary antibodies were stained with secondary antibodies (donkey-anti-rat-Alexa Fluor 488, polyclonal, Jackson ImmunoResearch, 712-545-153, 1:200; goat-anti-hamster-Cy3, polyclonal, Jackson ImmunoResearch 110,657, 1:200; goat-anti-rabbit-Alexa Fluor 647, polyclonal, Thermo Fisher, A27040, 1:200) for 1 h at RT. In parallel to the secondary antibody staining, nuclei were stained using DAPI (1:1000). After staining, tissue autofluorescence was blocked using TrueBlack lipofuscin (1× solution in 70% ethanol; biotium 23,007) incubation for 30 s. Starting from the antibody incubation, samples were washed after all steps 3× for 5 min with TBS/0.1% Tween or PBS (before mounting). The samples were mounted using a Fluoromount fluorescent mounting medium (Agilent Technologies, S302380–2) and stored at 4°C. Whole slide fluorescence images were acquired using the Slidescanner Pannoramic MIDI II (3D Histech). For the analysis of CD45- and CD3-positive areas, two 20× magnification fields of view were taken per sample using Slideviewer 2.5 (3D Histech). The images were processed using ImageJ Fiji 2.3.0,^56^ and the different stainings were segmented into objects using interactive machine learning within Ilastik 1.3.2.^57^ After segmentation, masks for the regions of interest (epithelium and lamina propria) were created for each image in ImageJ Fiji using the segmented EpCAM objects as markers for the epithelium and manual exclusion of the lumen and muscularis. Finally, the CD3- and CD45-positive areas within the epithelium and lamina propria and the total area of the epithelium and lamina propria were quantified in CellProfiler 4.2.1^58^ using the region of interest masks and the segmented CD3 and CD45 objects.

For hematoxylin and eosin staining, the intestinal cryosections were rehydrated in dH2O, placed in hematoxylin (Carl Roth) for 8 min, blued under running tap water for 30 min and placed in eosin G (Carl Roth) for 3 min. Finally, the sections were run through a rising alcohol line (1× 96% EtOH, 2 × 100% EtOH, 2× Xylol) and mounted with Eukitt Quick-hardening mounting medium (Sigma-Aldrich). Whole slide light microscopy images were acquired using the Slidescanner Pannoramic MIDI II (3D Histech) and processed using Slideviewer 2.5 (3D Histech).

### Microbiome analysis

Feces were collected from the animals during euthanization. The intestinal content was collected from different intestinal sections. Feces and intestinal content were immediately fresh-frozen on dry ice and kept at −80°C.

According to the manufacturer’s protocol, DNA was isolated from intestinal contents with the Quick-DNA Miniprep Kit (Zymo Research).

Absolute bacterial load was determined by qPCR of the 16S rRNA gene. Isolated DNA was amplified and detected with SYBR-Green (Applied Biosystems) in 96-well optical plates (Applied Biosystems) using the Applied Biosystems QuantStudio 5 system (Thermo Fisher Scientific) and universal primers (Univ 337 F 5’-ACTCCTACGGGAGGCAGCAGT-3’ and Univ 518 R 5’-GTATTACCGCGGCTGCTGGCAC-3’). For the amplification, 450 nM primers, 4 ng DNA, and 2× PowerUp™ SYBR™ Green Master Mix (Applied Biosystems) were used in a total volume of 5 µl. The amplification was performed using standard conditions (1 cycle 50°C 2 min, 1 cycle 95°C 2 min, 40 cycles of 95°C 15 s, 54°C 15 s, 72°C 1 min). The measurement was performed in triplicates with ROX as passive reference (Applied Biosystems). Standard curves were generated using a 10-fold serial dilution in the range of 10°-10^9^ copies of the 16S rRNA gene of *E. coli* (Invitrogen, C404010) amplified with primers 27 F (5′-GTTTGATCCTGGCTCAG-3′) and 1492 R (5′-CGGCTACCTTGTTACGAC-3′). Using the standard curve, 16S rDNA copy numbers per gram content were calculated.

The samples were stored at −20°C before being shipped on dry ice to Novogene (UK) for sequencing. DNA was fragmented and underwent end repair and phosphorylation. Next, A-tailing and adaptor ligation were performed. Finally, 150 bp paired-end sequencing was performed on the Illumina Novaseq 6000. Whole-genome shotgun sequencing data were processed using ngless (v1.3.0). Metagenomic reads were quality trimmed (phred score < 25), and resulting reads shorter than 45 bp were discarded. Filtered reads were mapped against the mouse genome (GRCm39 masked regions aligned to the Progenomes gene catalog). Taxonomic classification was assigned within ngless^59^ by aligning the reads to mOTUs taxonomy (mOTUs v2.6.0^60^) with default parameters. We chose this database to maximize the taxonomic assignment rate and minimize the impact of reference database incompleteness on our results, especially for the lower biomass small intestinal samples.

For functional profiling, trimmed and filtered reads were mapped against the mouse gut genes from the Global Microbial Gene Catalog (gmgc v1.0^61^) and summarized additionally on KEGG orthologs (KOs) and KEGG pathway levels via eggNOG annotations.

### Microbiome statistical analysis

#### Data preprocessing

The following steps were used to clean the taxonomic data at the mOTU level: (1) samples with low read counts (less than 500 reads) were discarded (Figure S5a, Additional File 1), and (2) mOTUs that were not present in at least 10% of the sample and accounted for at least 0.01% of the total abundance were discarded. Samples were not rarefied for any analysis, as preliminary alpha diversity analysis between rarefied and non-rarefied samples was comparable (Figure S5b, Additional File 1). In addition, two other samples were discarded because they had amplified the same mOTU detected in the blank sample (Figure S5c, S5d, S5e, Additional File 1). This resulted in the following samples of n numbers for the microbiome analysis: duodenum *n* = 2, jejunum *n* = 4, ileum *n* = 4, cecum *n* = 6, and colon *n* = 6. Figures that contain duodenum are only for descriptive purposes and were not statistically tested.

#### Analysis

Alpha diversity (~ Shannon index) and beta diversity were calculated on non-rarefied relative abundance mOTU-level data using the vegan package (v2.6.4). The Euler diagrams of the mOTU (~species), KO and GMGC levels were estimated (where the intersection was calculated based on the binary presence-absence status of each feature) and plotted using the Euler package (v7.0.0). The common/core mOTUs (~species) were also calculated using this binary present−absent approach. The overrepresentation test among different immune cell subset categories was performed using Fisher’s test. Differential heat trees were visualized using the metacoder (v0.3.7) package.

To model the association of immune cell subsets with colonization status (CONV or GF) and/or segment specificity, colonization status was recorded into a binary variable (0 (CONV), 1(GF)) and the segments into categorical variables. The relative abundance of each immune cell subset was then modeled individually by ordinary least square regression models against a colonization status-by-segment interaction term. The interaction-term tests for a significant association between the immune cell subset and colonization status, modified by the/a segment specificity (i.e., the effect of a segment on immune cell abundance is variable based on the colonization status). If a significant interaction was present, we grouped these populations under the category of “interaction”. In the absence of a significant interaction, we looked to see if both the main effects (colonization status and segment) were significant; if yes, those immune populations were grouped under the “main effects (without interaction)” category. If only one of the main effects was considered significant, those populations were categorized into “segment only” or “microbiome only”. The categories of the different heatmaps in Figure 5 correspond to this. Marginal mean estimates are shown for certain populations from these different categories overlaid on top of the raw data. The Benjamini‒Hochberg method (FDR < 0.05) was used to control the Type-1 error. Correlation analyses (Figure S4d) between microbial species and immune cell subsets was performed using both relative and absolute datasets. For each approach, the immune data were normalized to account for the effect of the germ-free (GF) condition. Specifically, the values for each immune cell population per sample were divided by the median value of the corresponding population in the GF group. This normalization step allowed for the assessment of the conventional (CONV) microbiome’s impact on immune cell populations relative to the GF baseline. The correlation analyses were performed across all intestinal segments without considering segment specificity.

The following R packages were used to model, check model diagnostics, and to tidy and visualize the final models: stats (v4.2.1), broomExtra (v5.0.0), performance (v0.10.8), and modelbased (v0.8.5).

### General statistical analysis

Unless otherwise stated, the Mann−Whitney U test was used to compare between groups and Benjamini‒Hochberg method (FDR < 0.05) was used to control Type-1 error.

## Supplementary Material

Supplemental Material

## Data Availability

The microbiome shotgun sequencing data supporting the conclusions of this article are available under the BioProject ID PRJNA1055712 from the NIH Sequence Read Archive (https://www.ncbi.nlm.nih.gov/sra/PRJNA1055712). The R code, processed sequencing data, and processed immune data used for statistical analysis and to generate the figures in this study are archived in Zenodo with the DOI: 10.5281/zenodo.10491380 (https://zenodo.org/doi/10.5281/zenodo.10491379). We provide all our comprehensive immune and microbiome data as an open-access app for exploration: https://wilck-lab.shinyapps.io/galt_2024_shiny/.
