# Supplementary material for: Segmental patterning of microbiota and immune cells in the murine intestinal tract
Source: Gut Microbes. 2024 Sep 10;16(1):2398126. doi: 10.1080/19490976.2024.2398126 (PMC11404582; doi:10.1080/19490976.2024.2398126)
Supplement: Supplemental Material [file KGMI_A_2398126_SM8584.zip › Additional_File_1 clean.docx]

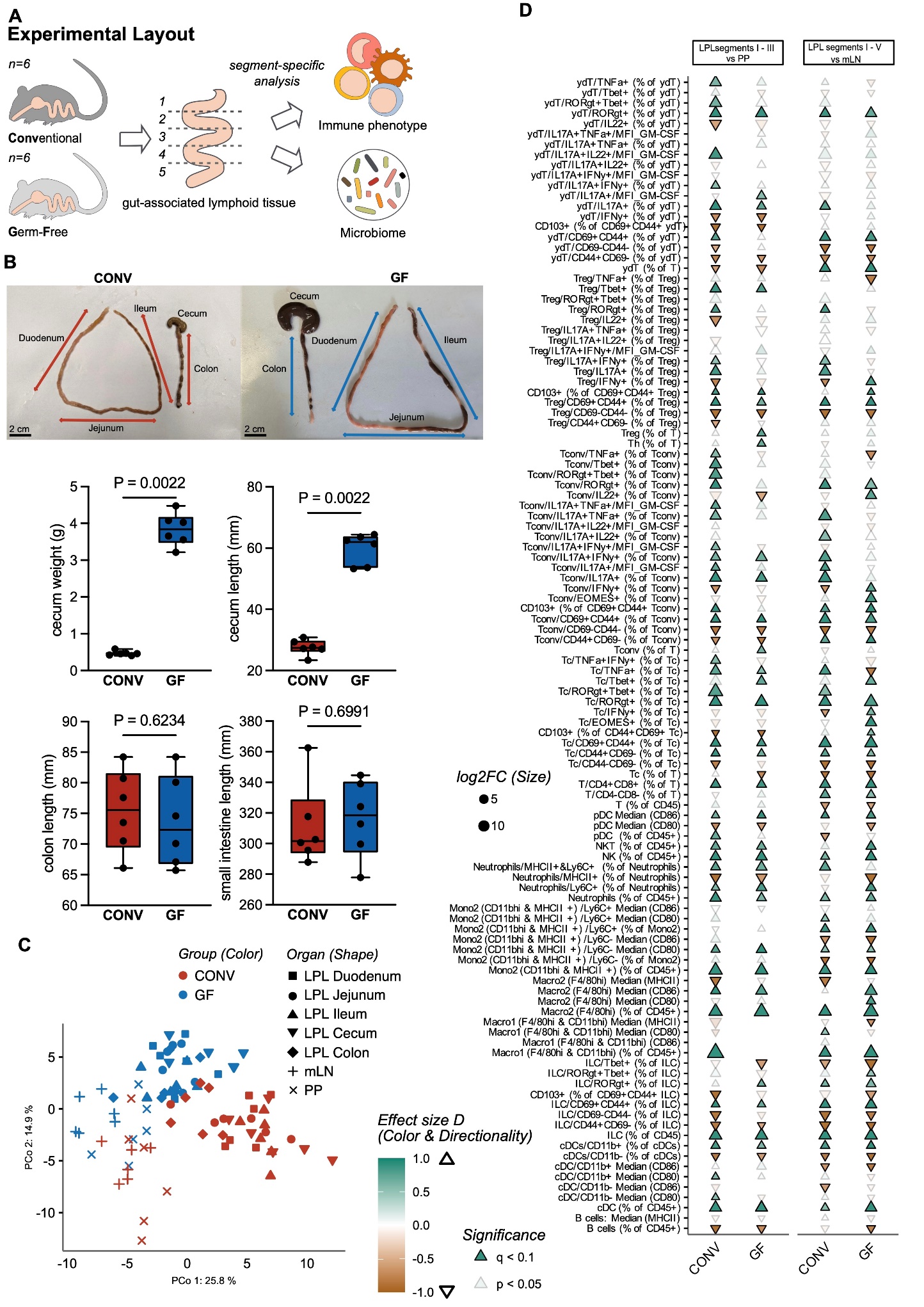


**Fig S1.** (A) Experimental design. (B) Representative photos of conventional (CONV) and germ-free (GF) mouse intestines and the segmentation approach (C) PCA from immune cell populations of the mesenteric lymph nodes (mLN), Peyer’s patches (PP) and lamina propria leukocytes (LPL) (D) Cuneiform plots show the comparison of small intestinal LPL with PP (left) and all LPL with mLN (right). Size of triangles correspond to the absolute effect size (Cliff’s delta) and transparency of triangles indicates significance (q<0.1 as solid shape, and p<0.05 (but not under q<0.1) as transparent) (n=6 mice per group).

**Fig S2: Morphological changes in the intestine between GF and CONV** (A) H&E- and immunofluorescence-stained sections of the different intestinal segments. Sections were stained for CD45 (lymphocytes, red), CD3 (T cells, green), EpCAM (epithelium, gray) and DAPI (cell nuclei, blue). Representative images of whole slides and 20x magnifications are shown. Scale bars equal 1000 µm (caecum whole slides), 500 µm (small intestinal, colon whole slides) or 200 µm (20x magnifications).(B) Quantitative analysis of the lamina propria and epithelial area based on EpCAM epithelial staining. P values were calculated using Mann-Whitney U test and BH-FDR correction (*q < 0.05) (n=6 mice per group)..


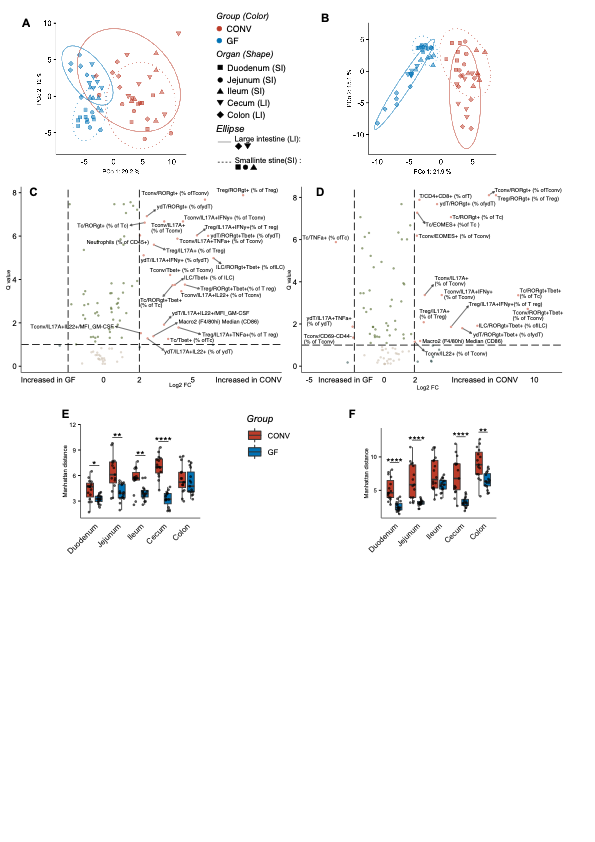
 **Fig S3: LPL and IEL are strongly influenced by colonization status.** PCA from the relative immune cell abundance in lamina propria leukocytes (LPL, A) and intraepithelial leukocytes (IEL, B). Volcano plots indicating immune cell populations enriched in germ-free (GF) mice (to the left) and conventional (CONV) mice (to the right) for LPL (C) and IEL (D). y-axis shows q-value, x-axis log-transformed fold change. Inter-sample distances for each LPL (E) and IEL (F) segment within CONV and GF mice. P values calculated using Mann Whitney U test (*** p < 0.001, ** p < 0.01, * p < 0.05) (n=6 mice per group)..


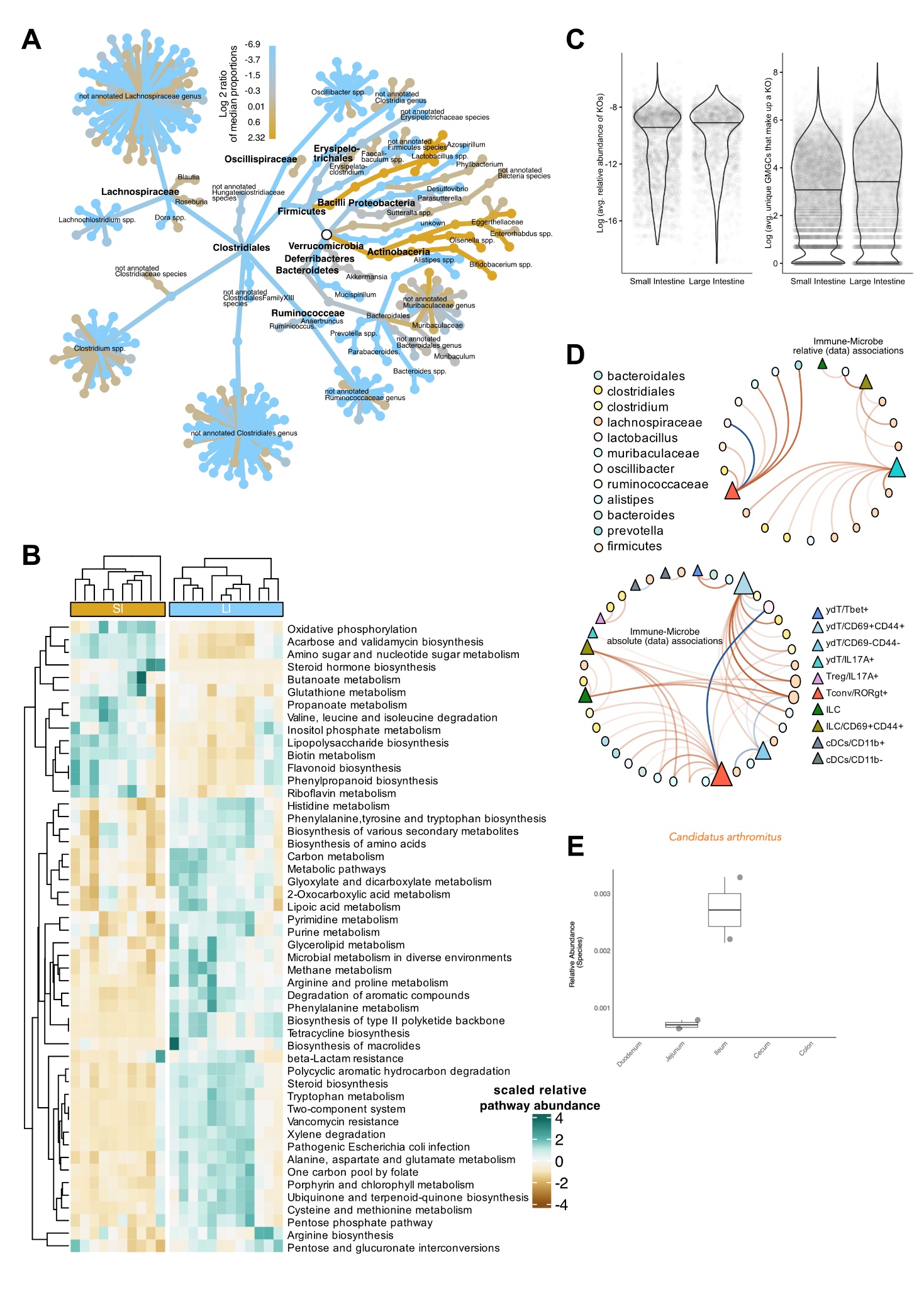


**Fig S4.** (A) Differential heat tree depicting the taxonomies of bacterial species that significantly differed in their relative abundance between small (n=10) and large intestinal (n=12) samples. Blue colored nodes show enrichment of those taxa in large intestine and orange in small intestine. (B) Heatmap showing the significantly (q<0.1) differentially regulated KEGG pathways between small and large intestinal samples. (C) Violin plot showing the distribution of relative abundance of each KO per sample (left) and the average number of unique GMGCs that make up each KO per sample (right). (D) Correlation analyses showing the spearman correlation coefficient between different immune cell subsets and microbial species between the relative (top) and absolute (bottom) data spaces. Species are colour-grouped to the next higher level with definitive taxonomic assignment. Colour of arc shows the direction of correlation (Blue: positive, Orange: Negative). (E). Relative abundance of the species *Candidatus arthromitus* from the samples in which it was detected.


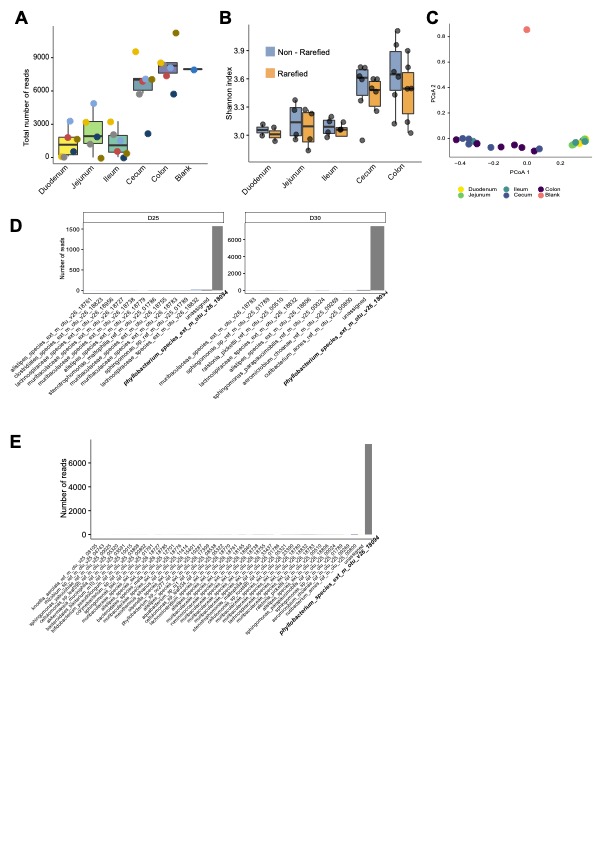


**Fig S5: Quality control of the microbiome analysis.** (A) Plots showing the total number of counts per sample (add all the counts across all the species), each colour represents an individual mouse. (B) Box plots comparing rarefied and non-rarefied alpha diversity (Shannon diversity metric). (C) Principal coordinate analysis on all samples, including blank, at the species level, using Euclidean distance. Note the clear separation of the blank samples from the rest. Relative abundance of the potential contaminant, *Phyllobacterium species*, in two samples (D) and in the blank (E).
