# Supplementary material for: Segmental patterning of microbiota and immune cells in the murine intestinal tract
Source: Gut Microbes. 2024 Sep 10;16(1):2398126. doi: 10.1080/19490976.2024.2398126 (PMC11404582; doi:10.1080/19490976.2024.2398126)
Supplement: Supplemental Material [file KGMI_A_2398126_SM8584.zip › Additional_File_2.docx]

**Table S1: Definition of the analyzed immune cell subsets**

| \| **Name (short)** \| **Name (full)** \| **Full marker definition** \| \| --- \| --- \| --- \| \| T \| T cells \| CD45+ CD3+ \| \| gdT \| Gamma delta T cells \| CD45+ CD3+ TCRgd+ \| \| Th \| T helper cells \| CD45+ CD3+ TCRgd- CD4+ CD8- \| \| Tconv \| Conventional T helper cells \| CD45+ CD3+ TCRgd- CD4+ CD8- FoxP3- \| \| Treg \| Regulatory T cells \| CD45+ CD3+ TCRgd- CD4+ CD8- FoxP3+ \| \| ILC \| Innate lymphoid cells \| CD45+ CD3- CD19- Gr-1- CD11b- EOMES- \| \| NKT \| Natural killer T cells \| CD45+ CD3+ NK1.1+ \| \| NK \| Natural killer cells \| CD45+ CD3- NK1.1+ \| \| Neutrophils \| Neutrophils \| CD45+ CD3- NK1.1- CD11b+ Ly6G+ SSC-A*high* \| \| B \| B cells \| CD45+ CD3- NK1.1- Ly6G- CD19+ \| \| cDCs \| Classical dendritic cells \| CD45+ CD3- NK1.1- Ly6G- CD19- CD45R- CD11c+ MHCII+ \| \| Macro1 \| Macrophage definition 1 \| CD45+ CD3- NK1.1- Ly6G- CD19- CD45R- CD11b+ F4/80*high* CD11b*high* \| \| Macro2 \| Macrophage definition 2 \| CD45+ CD3- NK1.1- Ly6G- CD19- CD45R- CD11b+ F4/80*high* CD11b*dim* \| \| Mono \| Monocyte definition 1 \| CD45+ CD3- NK1.1- Ly6G- CD19- CD45R- CD11b+ F4/80*low* CD11b*high* \| \| Mono2 \| Monocyte definition 2 \| CD45+ CD3- NK1.1- Ly6G- CD19- CD45R- CD11b+ F4/80- CD11b*high* MHCII+ \| \| pDC \|  \| CD45+ CD3- NK1.1- Ly6G- CD19- CD45R+ CD11b- Ly6C+ \| |
| --- | --- | --- | --- | --- | --- | --- | --- | --- | --- | --- | --- | --- | --- | --- | --- | --- | --- | --- | --- | --- | --- | --- | --- | --- | --- | --- | --- | --- | --- | --- | --- | --- | --- | --- | --- | --- | --- | --- | --- | --- | --- | --- | --- | --- | --- | --- | --- | --- | --- | --- | --- |

**Table S2: Antibodies used for immunophenotyping.**

| \| **Antibody** \| **Clone** \| **Company** \| **Cat No.** \| \| --- \| --- \| --- \| --- \| \| CD103 PE-Dazzle 594 \| 2E7 \| BioLegend \| 121430 \| \| CD115 APC \| REA827 \| Miltenyi Biotech \| 130-112-640 \| \| CD11b Alexa Fluor700 \| M1/70 \| BD pharmingen \| 557960 \| \| CD11b Biotin \| RB6-8C5 \| BioLegend \| 108403 \| \| CD11c PerCP-Cy5.5 \| N418 \| BioLegend \| 117327 \| \| CD127 BV711 \| A7R34 \| BioLegend \| 135035 \| \| CD19 APC-Vio770 \| REA749 \| Miltenyi Biotech \| 130-111-886 \| \| CD3 BV421 \| 17A2 \| BD Horizon \| 564008 \| \| CD4 APC-Vio700 \| GK1.5 \| Miltenyi Biotech \| 130-118-957 \| \| CD4 BV711 \| RM4-5 \| BioLegend \| 100549 \| \| CD44 FITC \| IM7 \| BD pharmingen \| 553133 \| \| CD45 PE Vio770 \| REA737 \| Miltenyi Biotech \| 130-110-661 \| \| CD45R Biotin \| RA3-6B2 \| BioLegend \| 103203 \| \| CD45R PE-Cy5 \| RA3-6B2 \| BioLegend \| 103210 \| \| CD69 PE-Cy5 \| H1.2F3 \| BioLegend \| 104510 \| \| CD8a BV650 \| 53-6.7 \| BioLegend \| 100741 \| \| CD8a PerCP Cy5.5 \| 53-6.7 \| invitrogen \| 45-0081-82 \| \| CD80 BV650 \| 16-10A1 \| BioLegend \| 104732 \| \| CD86 BV711 \| GL1 \| BD OptiBuild \| 740688 \| \| EOMES APC eFluor 780 \| WD1928 \| eBioScience \| 47-4877-41 \| \| F4/80 PE \| BM8 \| BioLegend \| 123110 \| \| Fc Block \|  \| Miltenyi Biotech \| 130-092-575 \| \| FoxP3 Alexa Fluor 700 \| FJK-16s \| eBioScience \| 56-5773-82 \| \| GM-CSF PE-Dazzle594 \| MP1-22E9 \| BioLegend \| 505421 \| \| Gr-1 Biotin \| RB6-8C5 \| BioLegend \| 108403 \| \| IFNg BV785 \| XMG1.2 \| BioLegend \| 505838 \| \| IL-17A PE \| eBio17B7 \| eBioScience \| 12-7177-81 \| \| IL-22 PerCP eFluor 710 \| IL22JOP \| life technologies \| 46-7222-82 \| \| Ly6C BV605 \| AL-21 \| BD Horizon \| 563011 \| \| Ly6G PE-Vio615 \| REA526 \| Miltenyi Biotech \| 130-123-029 \| \| MHCII BV785 \| M5/114.15.2 \| BioLegend \| 107645 \| \| NK1.1 VB-B515 (FITC) \| REA1162 \| Miltenyi Biotech \| 130-120-503 \| \| RORgt BV650 \| Q31-378 \| BD Horizon \| 564722 \| \| Streptavidin BV785 \|  \| BioLegend \| 405249 \| \| Streptavidin PE-Cy5 \|  \| BioLegend \| 405205 \| \| Tbet PE \| REA102 \| Miltenyi Biotech \| 130-121-340 \| \| TCRgd BV605 \| GL3 \| BioLegend \| 118129 \| \| TNFa FITC \| REA636 \| Miltenyi Biotech \| 130-124-212 \| |
| --- | --- | --- | --- | --- | --- | --- | --- | --- | --- | --- | --- | --- | --- | --- | --- | --- | --- | --- | --- | --- | --- | --- | --- | --- | --- | --- | --- | --- | --- | --- | --- | --- | --- | --- | --- | --- | --- | --- | --- | --- | --- | --- | --- | --- | --- | --- | --- | --- | --- | --- | --- | --- | --- | --- | --- | --- | --- | --- | --- | --- | --- | --- | --- | --- | --- | --- | --- | --- | --- | --- | --- | --- | --- | --- | --- | --- | --- | --- | --- | --- | --- | --- | --- | --- | --- | --- | --- | --- | --- | --- | --- | --- | --- | --- | --- | --- | --- | --- | --- | --- | --- | --- | --- | --- | --- | --- | --- | --- | --- | --- | --- | --- | --- | --- | --- | --- | --- | --- | --- | --- | --- | --- | --- | --- | --- | --- | --- | --- | --- | --- | --- | --- | --- | --- | --- | --- | --- | --- | --- | --- | --- | --- | --- | --- | --- | --- | --- | --- | --- | --- | --- | --- | --- | --- | --- | --- |
